# Supplementary material for: Asymmetric Synthesis of Quaternary Hydantoins via a Palladium-Catalyzed Aza-Heck Cyclization
Source: J Am Chem Soc. 2025 Nov 14;147(49):44692–8. doi: 10.1021/jacs.5c16022 (PMC12703750; doi:10.1021/jacs.5c16022)
Supplement: Supplementary file 2 [file ja5c16022_si_002.zip › All NMR FID Files/S50/S50_CarbonNMR/3/pdata/1/email_EM05-183-2-13C_3_1.pdf]

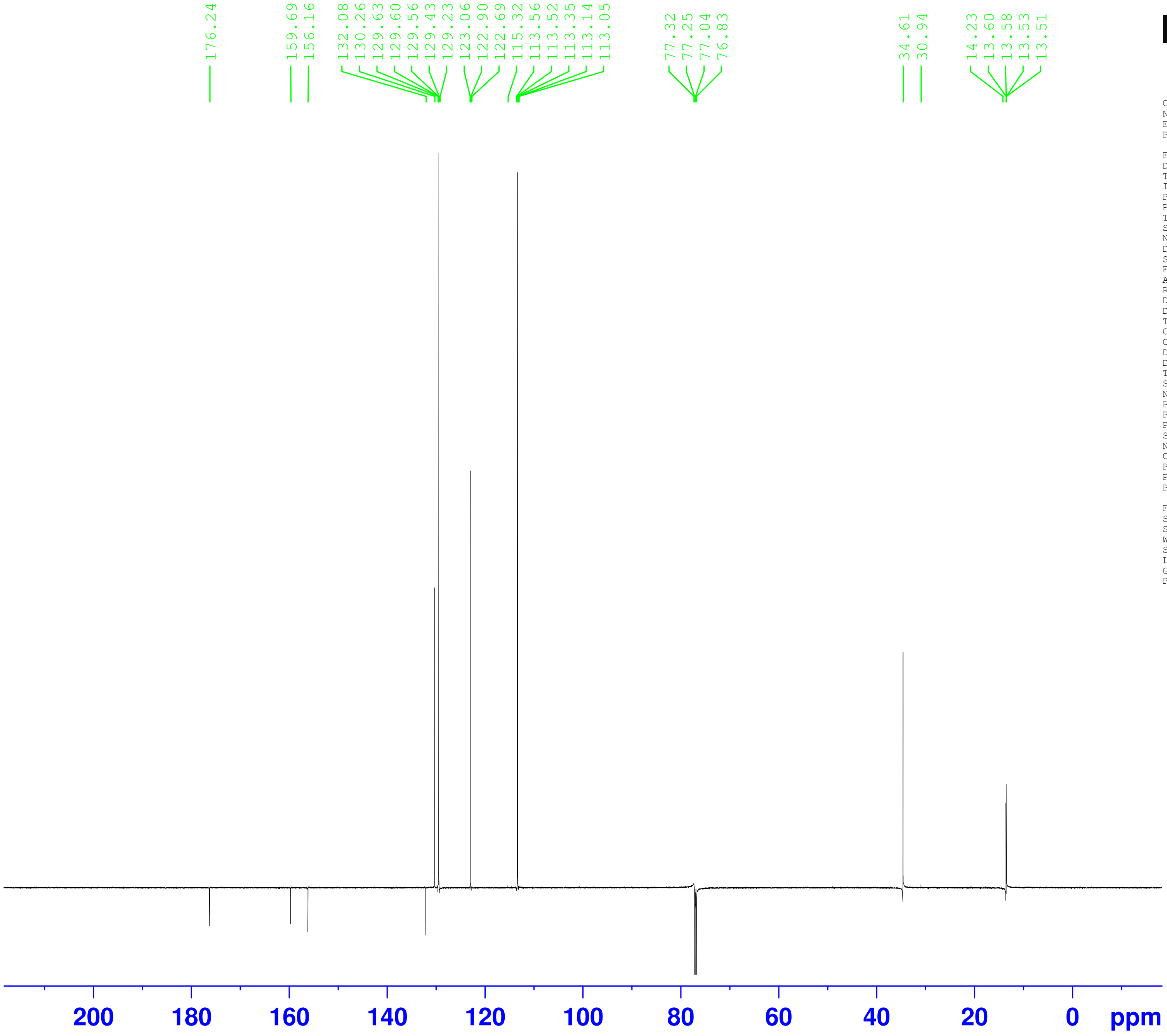


```
Current Data Parameters
NAME      EM05-183-2-13C
EXPNO                  3
PROCNO                 1

F2 - Acquisition Parameters
Date_           20240816
Time               18.44 h
INSTRUM  Avance Neo 600 at University of Delaware
PROBHD   Z185170_0001 (
PULPROG             jmod
TD                 65536
SOLVENT            CDCl3
NS                  1024
DS                     4
SWH            35714.286 Hz
FIDRES          1.089913 Hz
AQ             0.9175040 sec
RG                   101
DW                14.000 usec
DE                 19.25 usec
TE                 298.0 K
CNST2        145.0000000
CNST11         1.0000000
D1            2.00000000 sec
D20           0.00689655 sec
TD0                    1
SFO1         150.9178988 MHz
NUC1                 13C
P1                 11.98 usec
P2                 23.96 usec
PLW1        106.00000000 W
SFO2         600.1324005 MHz
NUC2                  1H
CPDPRG[2         waltz65
PCPD2              70.00 usec
PLW2          6.90000010 W
PLW12         0.09399300 W

F2 - Processing parameters
SI                 32768
SF           150.9028085 MHz
WDW                   EM
SSB                    0
LB                  1.00 Hz
GB                     0
PC                  1.40
```
